# Early ERP processing of self-referential language processing in voluntary and involuntary action contexts: N170 and EPN

**DOI:** 10.1007/s00426-026-02370-x

**Published:** 2026-10-29

**Authors:** Joanna Daria Dołżycka, Cornelia Herbert

**Affiliations:** Department of Applied Emotion and Motivation Psychology, Ulm University, Albert- Einstein-Allee 47, Ulm, 89081 Germany

## Abstract

Understanding how the brain differentiates self from others through language represents an important topic in psychology, linguistics, and social cognition. Within an embodied cognition framework, the present EEG study investigated early neural responses to isolated German pronouns varying in self-relatedness (self-, other-, and self/other-related) and grammatical type (personal vs. possessive). Forty right-handed native German speakers silently read visually presented pronouns while EEG activity was recorded. Two task contexts were employed, involving voluntary and involuntary keypress responses following stimulus presentation. Event-related potential (ERP) analyses revealed significant modulations of the N170 and Early Posterior Negativity (EPN) components. N170 amplitudes varied as a function of pronoun type and self-relatedness, with self-related and other-related pronouns eliciting different responses from ambiguous second-person forms. EPN amplitudes differed across self-reference categories, with additional interactions involving hemisphere and pronoun type. Together, these findings suggest that even minimal linguistic stimuli such as pronouns engage early neural processes sensitive to self-relatedness and linguistic context. Task conditions also differed in early ERP amplitudes; because responses in the immediate-response condition overlapped the ERP measurement intervals, these differences are reported without functional attribution.

## Introduction

Understanding how individuals conceptualize themselves and distinguish self from others is a core topic in cognitive neuroscience and psycholinguistics. This study approaches the issue from the perspective of embodied cognition, which posits that cognitive processes fundamentally arise from sensory-motor interactions and bodily experiences, rather than abstract symbolic representations. Language, particularly pronouns, serves as a critical medium for expressing self-concepts and mediating social interactions. Pronouns not only linguistically encode distinctions between self and other but also reflect deeper cognitive and emotional frameworks. Since the choice of ‘I’ versus ‘my’ or ‘you’ versus ‘your’ reflects patterns of self-construal, ownership, and interpersonal distance (Olekalns et al., 2010; Olekalns & Druckman, 2014; Kross et al., 2014), these distinctions can shape how individuals perceive themselves and relate to others (Blume & Herbert, 2014; Herbert et al., 2016). This investigation integrates embodied cognition with linguistic and neuroscientific approaches to explore how pronoun processing is linked to self-other differentiation in the brain.

### Embodied cognition and self-other differentiation

Embodied cognition proposes that the mind is inherently tied to the body, and that cognitive processes are deeply embedded in physical actions and sensorimotor interactions (Varela et al. 2017; Johnson and Lakoff 2002a, b). This perspective challenges traditional models based on abstract symbolic processing and emphasizes the inseparability of mind, body, and environment (DeSutter & Stieff, 2017; Adams, 2010). Recent neural evidence supports this view, particularly through studies employing event-related potentials (ERPs). The N170 component, which is sensitive to facial recognition and emotional expression, reflects the rapid perceptual and emotional appraisal associated with socially meaningful stimuli (Hinojosa et al., 2015; Calvo & Nummenmaa, 2016). Calvo and Nummenmaa (2016) note that the N170’s sensitivity depends not only on the emotional content of expressions but also on the perceptual salience of facial features, illustrating its central role in fast, adaptive social perception. Complementing this, Hinojosa et al. (2015) demonstrated that emotional facial expressions reliably enhance N170 amplitudes, further underscoring the component’s relevance for processing socially salient cues. Additionally, Cygan et al. (2014) found lateralized N170 patterns for self-related stimuli—names elicited stronger left-hemisphere responses, whereas one’s own face produced heightened right-hemisphere amplitudes—highlighting the N170’s sensitivity to distinctions between self and other.

The Early Posterior Negativity (EPN) component has been linked to early semantic processing (Kissler et al., 2007; Herbert et al., 2008) and facilitated emotional evaluations of self-referential emotional pronoun-noun pairs (e.g., Herbert et al., 2011), and this modulation might be driven by embodied experiences (Mavratzakis, 2016; Herbert, 2023; for a discussion). Building on this, the EPN, occurring shortly after the N170, might index deeper emotional and cognitive processing modulated by embodied factors (Farkas & Sabatinelli, 2023; Liu et al., 2024). Additional behavioral work supports this connection: embodied actions such as gestures and movements enhance learning and emotional engagement (DeSutter & Stieff, 2017), and virtual reality studies demonstrate that immersive bodily interactions elicit stronger cognitive involvement (Kim et al., 2014). Emotional cognition is also deeply informed by interoceptive signals, which integrate bodily states with psychological experiences (Seth, 2013; Seth & Friston, 2016). Taken together, these findings converge to show that both cognitive and emotional processes are grounded in physical and bodily experience.

In addition to their well-established roles in face and emotion perception, both the N170 and EPN have also been implicated in the processing of written linguistic stimuli. Early visual ERP components are sensitive to lexical properties such as word length and frequency (Hauk & Pulvermüller, 2004), indicating that linguistic information is rapidly evaluated at perceptual stages. Crucially, these components are modulated by the social and emotional relevance of words: self-referential linguistic stimuli elicit enhanced perceptual and affective responses even in the absence of explicit self-appraisal instructions (Herbert et al., 2011). Additionally, N170-like responses have been reported for one’s own name and other self-related words; for example, Cygan et al. (2014) demonstrated that N170 amplitudes can differentiate between hearing one’s own name versus others’ names, and between viewing one’s own face versus a familiar face. Such findings show that the N170 and EPN are responsive not only to visual and emotional stimulus properties but also to the social-cognitive meaning embedded in language. Because pronouns constitute minimal yet powerful linguistic markers of self and other, they are well positioned to engage these early differentiation mechanisms. This provides a strong theoretical basis for expecting N170 and EPN modulations during pronoun processing, similar to effects observed for self-related words and names.

Together, these ERP findings highlight how both early perceptual mechanisms (captured by the N170) and later emotional-evaluative mechanisms (captured by the EPN) reflect embodied self–other processing (Blume & Herbert, 2014; Herbert et al., 2016). This provides a theoretical foundation for examining whether linguistic cues such as pronouns—minimal but inherently self- or other-referential stimuli—can also engage these embodied differentiation mechanisms.

### Pronouns as linguistic, emotional and cognitive markers

Linguistically, pronouns function as deictic or indexical markers: their reference shifts depending on the speaker, addressee, and discourse context. Unlike nouns, which have stable referents, pronouns derive their meaning from situational cues and social roles, making them highly sensitive to perspective-taking (e.g., Levinson, 2007). Personal pronouns encode grammatical person (first, second, third), while possessive pronouns additionally encode ownership relations, marking distinctions between identity and possession. These linguistic features make pronouns uniquely suited for probing self–other differentiation. First-person pronouns (‘I’, ‘me’) are closely linked to self-awareness and personal identity, particularly during heightened self-attention (Hirschfeld, 1996; Silvia, 2002; Tagini & Raffone, 2010). They also capture cultural variations in self-concept: individualistic cultures tend to employ first-person singular forms to emphasize autonomy, whereas collectivist cultures rely more on inclusive forms such as ‘we’ to foreground community identity (Oyserman et al., 2002; Kitayama et al., 2007). From a cognitive-emotional perspective, third-person pronouns (‘he’, ‘she’) promote psychological distancing and support theory-of-mind processes, aiding emotion regulation and perspective-taking (Bremner, 2013; Dolcos & Albarracín, 2014; Kross et al., 2014). Possessive pronouns (‘my’, ‘your’) highlight ownership and interpersonal dynamics, playing key roles in contexts such as negotiation and conflict resolution (Olekalns et al., 2010; Olekalns & Druckman, 2014). Second-person pronouns (‘you’) directly engage interlocutors and contribute to shared emotional experiences (Fineberg et al., 2016; Zimmermann et al., 2017). Importantly for the present study, second-person pronouns were classified as self/other-related even when presented in isolation. Although no interlocutor was present during EEG recording, research shows that ‘you’ automatically evokes an implicit speaker–listener framework. The cognitive system simulates communicative roles even without social context, such that second-person forms trigger both self-relevant processing (as the potential addressee) and other-related processing (as the implied speaker). This automatic addressivity makes second-person pronouns inherently dynamic: they recruit self-focused attention while simultaneously invoking social perspective-taking. Thus, even in a decontextualized setting, second-person pronouns engage neural mechanisms related to self–other mapping. Neuroscientific research supports this interpretation, showing that self-referential pronouns activate medial prefrontal regions involved in self-processing and emotional evaluation (Blume & Herbert, 2014; Herbert et al., 2016). Pronoun usage also correlates with psychological traits and clinical conditions (Carey et al., 2015; Edwards & Holtzman, 2017; Kacewicz et al., 2014): for example, frequent use of first-person singular pronouns is associated with depression and anxiety (Brockmeyer et al., 2015; Tackman et al., 2019; Chen et al., 2024), while atypical pronoun patterns in autism (e.g., pronoun reversals) reveal challenges in self–other distinction and social cognition (Naigles et al., 2016; Overweg et al., 2018). Cross-linguistic research further shows that pronouns support flexible navigation of multiple cultural and linguistic identities (Alkhatib & Altakhaineh, 2023; Zhai & Fan, 2024). Finally, the grammatical distinction between personal and possessive pronouns is theoretically consequential for early ERP responses. Personal pronouns refer directly to agents (‘I’, ‘he’, ‘she’), whereas possessive pronouns encode ownership and relational boundaries (‘my’, ‘your’). These differences correspond to distinct social-cognitive functions—identity vs. ownership—and are known to engage early perceptual and affective processing systems. Research demonstrates that components such as the N170 and EPN are sensitive to socially meaningful linguistic cues even when stimuli differ in low-level features such as length or phonological complexity. Thus, differences between personal and possessive forms may manifest in early ERP components not because of word length alone, but because the two categories convey fundamentally different social-emotional meanings. Taken together, these findings indicate that pronouns function not only as grammatical markers but also as indicators of cognitive and emotional processes rooted in bodily and social experience.

### Aim of the present study

The present EEG study investigates the neural processing of isolated German pronouns within the embodied cognition framework and, importantly, examines whether early ERP responses to pronouns differ across two task contexts with different response requirements. To address this, the study uses a version of the HisMine paradigm (for an overview e.g., Herbert, 2022) and examines whether early ERP responses under conditions of voluntarily initiated pronoun presentation versus externally cued (involuntary) pronoun presentation. This manipulation varied response requirements and response timing across two task contexts, one in which the response was externally cued and one in which its timing was self-selected. This standpoint is grounded in embodied cognition theories suggesting that cognitive processes differ when individuals actively engage with stimuli rather than passively experience them (Stocker, 2015; Stegmann, 2020), as well as in neuroscientific accounts of agency showing that voluntary action relies on distinct monitoring and predictive mechanisms in the brain (Haggard, 2017). We expected that the two task contexts might differ in the balance of stimulus-related, motor-related, and preparatory processing. In line with these perspectives, we hypothesized that the externally cued condition may differentially influence early ERP responses due to differences in anticipatory, attentional, and response-related processing. (Eimer et al., 2009). As part of the larger project, the present report focuses on stimulus-locked ERPs elicited by pronouns. The present report examines whether pronoun self-relatedness, grammatical type, hemisphere, and task context are associated with early ERP differences. Therefore, the analysis of ERPs elicited by pronouns is reported in this manuscript separately and independently from the study of response-context and ownership-related effects, which would epoch the ERP processing differently. Within the current design, the study tests the hypothesis that self-referential first-person pronouns and other-referential third-person pronouns elicit distinct ERP patterns associated with pronoun self-relatedness and grammatical type. Additionally, second-person pronouns (e.g., ‘you’, ‘your’) are treated as self/other-related due to their dynamic reference frame, which recruits both self-perception and social perspective-taking. As personal pronouns directly reference entities (self or others as actors/agents), whereas possessive pronouns reference relationships of ownership or association we posit this grammatical distinction could carry social-cognitive significance even at early processing stages. For instance, possessive pronouns might automatically trigger a sense of ownership or territory (e.g., ‘my’/’your’ implying possession) and thus engage additional evaluative processes about self-other boundaries in a different way than simple personal references. In line with these considerations, we predicted that the N170 would exhibit enhanced amplitudes for self-related and second-person pronouns, reflecting increased salience for socially meaningful stimuli. For the EPN, we expected stronger responses to possessive pronouns due to their inherent links to ownership and interpersonal boundaries, as well as to self-related pronouns more broadly. Finally, we anticipated that ERP amplitudes might differ between the involuntary and voluntary task conditions, potentially reflecting differences in response preparation, anticipation, attentional engagement, and other task-related processes. Given the established sensitivity of the N170 and EPN components to social, emotional, and embodied cues, these ERP markers are used to probe the neural mechanisms underlying pronoun processing, with pronouns serving as minimal units of self–other recognition. Hemispheric specialization is also considered, particularly the roles of the right hemisphere in emotional-social processing and the left hemisphere in linguistic encoding (Tekülve & Schöner, 2024). By integrating task context, embodiment, and linguistic self-processing, the study examines how early ERP responses to pronouns differ across voluntary and involuntary response contexts, while recognizing that the specific contribution of agency cannot be isolated from response-related differences in the present design. In doing so, it seeks to advance theories of selfhood, cognition, and emotion and contributes to understanding how language and bodily experience interact to shape self-perception and interpersonal dynamics, with implications for therapeutic contexts in which pronoun usage reflects underlying emotional states.

## Materials and methods

### Participants

Forty right-handed native German speakers (32 women, 8 men; mean age = 22.40 years, SD = 3.12, range = 18–32) participated in the study. Participants were recruited via university flyers, academic email systems, and social media platforms, targeting both students and professionals. Each participant provided written informed consent prior to the experiment. They received either 25€ or 2.5 academic credits as compensation. The study was approved by the local ethics committee at Ulm University (Ethikkommission der Universität Ulm; https://www.uni-ulm.de/en/einrichtungen/ethikkommission-der-universitaet-ulm/), and all procedures were conducted in accordance with ethical guidelines. Participant data were anonymized to ensure confidentiality.

### Procedure

**Fig. 1:**
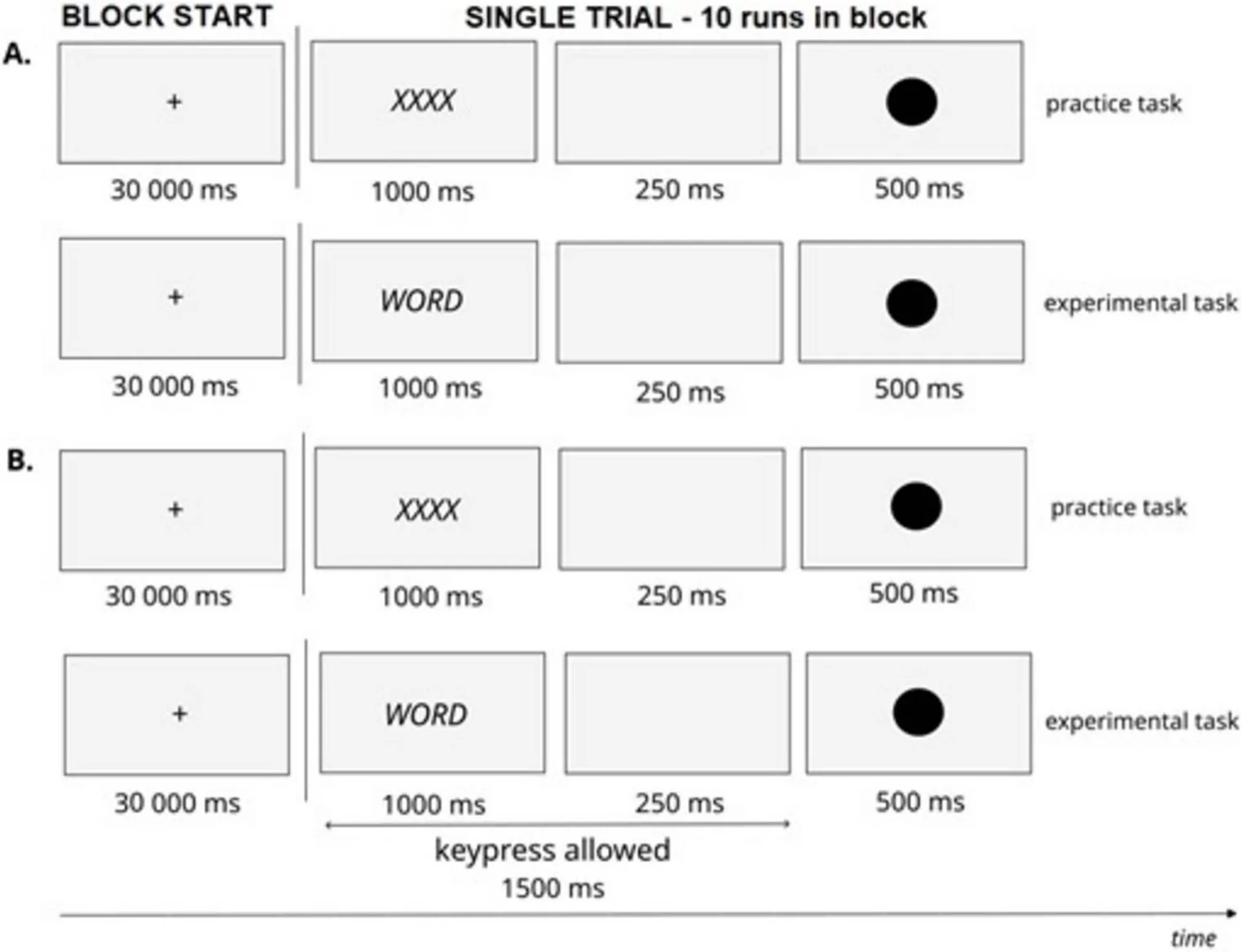
Scheme of the experimental procedure. (**A**) Involuntary (forced) keypress condition – participants must press the key immediately upon pronoun onset. (**B**) Voluntary keypress condition – participants initiate the keypress at a self-chosen time after the pronoun onset (before a response deadline indicated by a circle)

During the EEG experiment, stimuli were presented using PsychoPy v.2024.1.1 (Peirce, 2009) on a computer screen displaying white text on a gray background. Participants, seated comfortably, were instructed to silently read visually presented German pronouns, which comprised both personal (ICH [I], DU [you], ER [he], SIE [she]) and possessive forms (MEIN [my], DEIN [your], SEIN [his]). Each trial began with a 1000-ms presentation of a pronoun, followed by a 250‐ms blank screen and a 500‐ms display of a white circle indicating the response window. The experimental design featured two task conditions: in the involuntary (forced) keypress condition, participants were required to press a designated key immediately upon stimulus onset, whereas in the voluntary condition, participants pressed the key at a self‐selected time after the pronoun presentation but before the circle appeared. The experiment comprised 1400 trials in total, with 100 presentations per pronoun within each task condition. The German pronoun stimuli necessarily differ in physical features such as letter count and orthographic complexity, reflecting the natural variation found in the language. We retained this full range of pronoun forms to avoid artificially homogenizing the stimulus set, while acknowledging that this introduces some low-level variability within our isolated-word paradigm. This approach allows us to examine neural responses to self-referential and other-referential pronouns within a controlled yet linguistically grounded set of stimuli. Prior to the main experiment, participants completed practice trials using a row of the letter *X* that mimicked the timing and structure of the experimental runs, thereby ensuring task familiarization without introducing lexical confounds. The scheme of the procedure is presented in Fig. 1.

### Apparatus

For recording EEG activity during the task, we used BrainVision BrainAmp DC amplifier (Brain Products GmbH, Munich, Germany). For acquisition of scalp electrical activity, we used sixty-four active ag/agCl electrodes according to the international 10–20 system using an actiCAP system (Brain Products GmbH, Munich, Germany). We recorded electrooculogram (EOG) data, such as eye blinks and horizontal eye movements, using four active ag/agCl electrodes. Impedances were kept below 10 kΩ. Raw EEG signals were recorded at a sampling rate of 1000 Hz.

### EEG Data preprocessing and statistical analysis

**Fig. 2:**
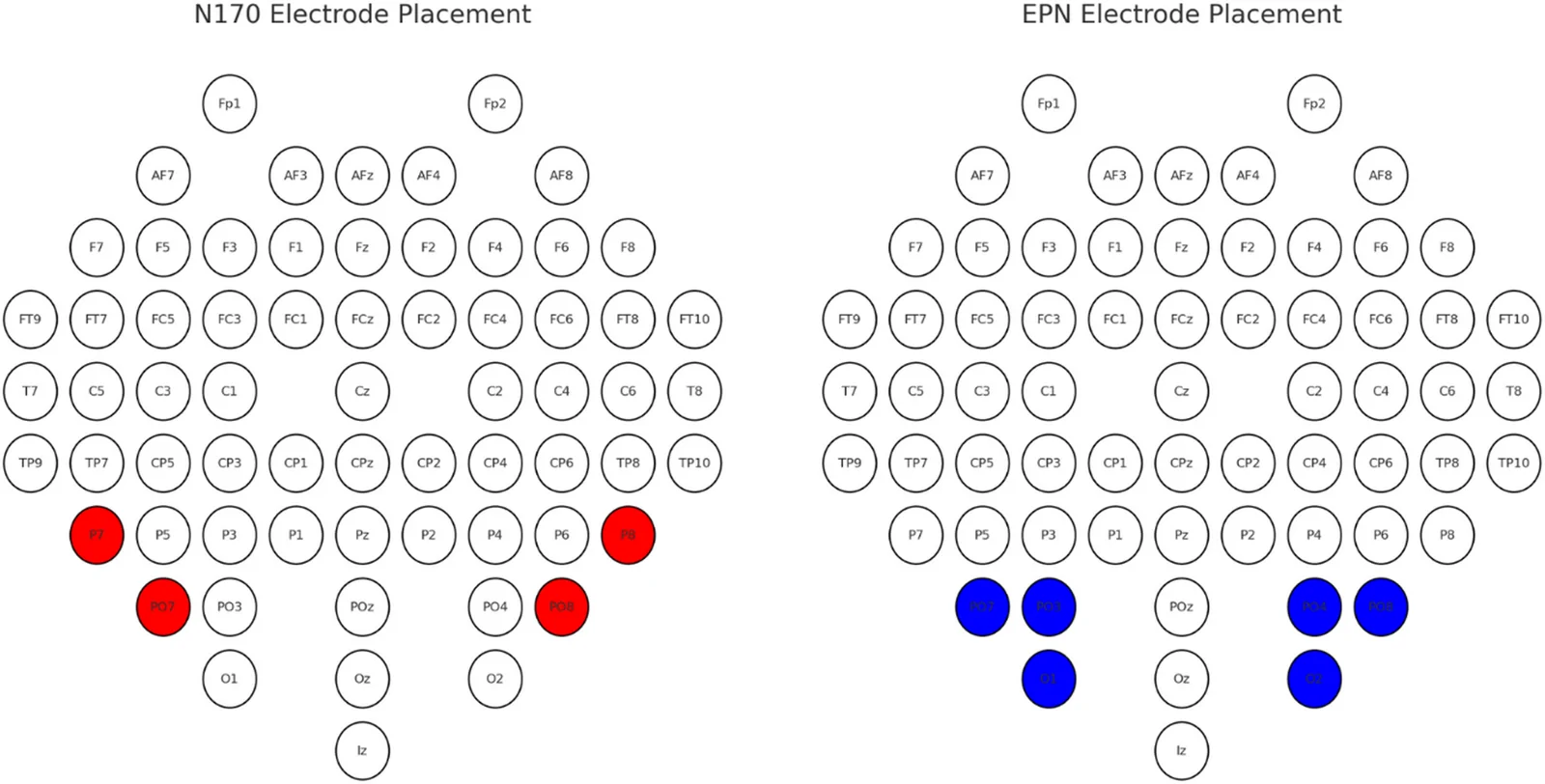
Electrode selection for N170 and EPN ERP components

EEG data were preprocessed using Brain Vision Analyzer 2 (Version 2.2.2; Brain Products GmbH, Giltching, Germany). Continuous data acquired at 1000 Hz were down-sampled to 256 Hz and band-pass filtered with cutoff frequencies of 0.15 Hz (low) and 30 Hz (high), with a 50 Hz notch filter applied. Ocular artifacts were corrected using an independent component analysis (ICA)–based algorithm; vertical eye movements were monitored via vertical electrooculogram (VEOG) channels (VEOG1 referenced to VEOG2) and horizontal movements via horizontal electrooculogram (HEOG) channels (HEOG1 and HEOG2). To enable effective artifact rejection—which is available only for segmented data—the EEG was initially segmented into extended epochs spanning − 400 to 1900 ms relative to word onset. This extended window served as a “shortcut” to capture artifacts occurring both before and after stimulus presentation, thereby maximizing the detection of contaminated segments. Following artifact rejection, the epochs were shortened by re-segmenting the data into a narrower window (–100 to 800 ms) that encompassed the event-related potential (ERP) components of interest, ensuring that subsequent analyses were conducted on clean data while preserving the critical temporal information. Artifact rejection was performed using semi-automatic raw-data inspection in BrainVision Analyzer 2, following standard ERP preprocessing criteria. The criteria included thresholds for excessive voltage steps, absolute amplitude, within-epoch voltage differences, and abnormally low activity. Baseline correction was applied using the − 100 to 0 ms pre-stimulus interval. Following artifact rejection, sufficient numbers of segments remained for ERP averaging across all participants and conditions. Contaminated channels were identified and reconstructed using triangulation and linear interpolation prior to rejection, with between 1 and 13 of 64 channels interpolated per participant (M = 2.90 channels, SD = 2.89). We specifically targeted ERP components: the N170, commonly associated with early stages of visual cognitive processing, and the Early Posterior Negativity (EPN), related to enhanced visual processing of emotionally or socially salient stimuli. For each ERP component, data were initially selected and averaged across electrode clusters, which were predefined based on established electrode placements in neuropsychological research and prior ERP literature (e.g., Wronka & Walentowska, 2011; Tanaka & Jiang, 2024; Gülbetekin et al., 2022; Kulke et al., 2022; Tanaka & Jiang, 2024). Specifically, for the analysis of the N170 component, electrodes located at lateral occipital and posterior-temporal regions (P7, P8, PO7, PO8) were selected, as these regions typically exhibit maximal amplitudes for this component. Posterior-occipital electrodes (PO3, PO4, PO7, PO8, O1, O2) were selected for analyzing the EPN component, reflecting the known scalp distribution patterns documented in previous ERP studies (Fig. 2). Both components were quantified over posterior electrode clusters rather than over central motor sites, where hand-related motor activity would generally be expected to be most pronounced. The N170 component was quantified as the mean amplitude in the 120–190 ms post-stimulus interval, and the EPN in the 200–300 ms interval, based primarily on prior ERP literature examining these components (e.g., Herbert et al., 2011; Hinojosa et al., 2015; Luck & Gaspelin, 2017). Visual inspection of the grand averages was used only as a secondary confirmation step.

### Statistical analysis

An omnibus repeated-measures ANOVA (rmANOVAs) was first conducted to identify significant main effects and interactions. The analyses were implemented utilizing Python programming language (Python version 3.11), using two statistical libraries known for their reliability and extensive use in psychological and cognitive research: ‘statsmodels’ (version 0.14.0; Seabold & Perktold, 2010) for running the repeated-measures ANOVAs, and ‘pingouin’ (version 0.5.3; Vallat, 2018) for conducting planned pairwise post-hoc comparisons. Four within-subject factors were explicitly considered in these statistical calculations: task condition (BLOCK: voluntary vs. involuntary keypress), hemisphere (LATERALITY: left vs. right hemisphere), pronoun type (PRONOUN: personal vs. possessive pronouns), and self-reference condition (SELF: self-related, other-related, and self/other-related pronouns). To ensure a more robust interpretation of the results, significant main effects and interactions identified by the omnibus ANOVA were further explored using detailed pairwise comparisons, with Bonferroni corrections applied within each effect to account for multiple testing and to control the Type I error rate, the number of comparisons being determined by the number of levels involved. Furthermore, effect sizes (Hedges’ g) quantified the magnitude of ERP differences. This comprehensive statistical approach allowed for identification of significant effects and interactions among experimental factors, enhancing the robustness and interpretability of ERP findings related to self-referential processing.

## Results of EEG analysis

**Fig. 3:**
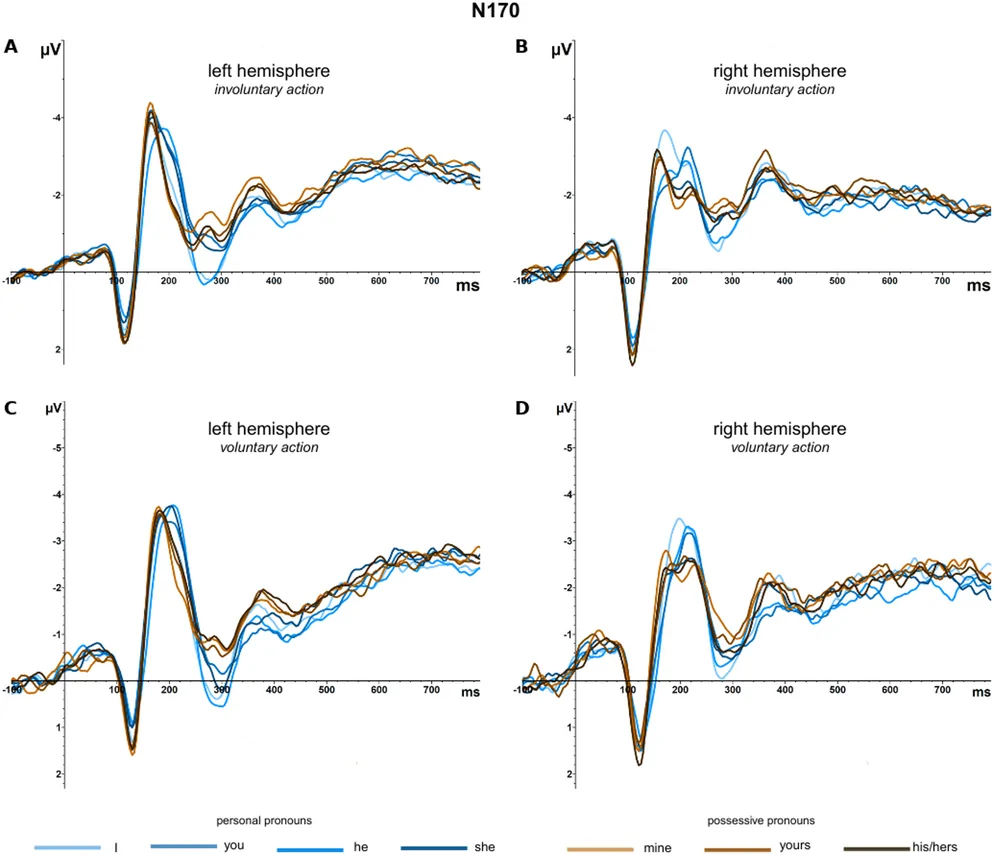
Grand-average N170 waveforms elicited by German personal and possessive pronouns at posterior electrode sites, shown separately for the involuntary and voluntary keypress conditions. Waveforms were averaged across the N170 electrode cluster and are displayed separately by hemisphere (left: P7, PO7; right: P8, PO8). (**A**) Left hemisphere, involuntary (forced) keypress. (**B**) Right hemisphere, involuntary (forced) keypress. (**C**) Left hemisphere, voluntary keypress. (**D**) Right hemisphere, voluntary keypress

**Fig. 4:**
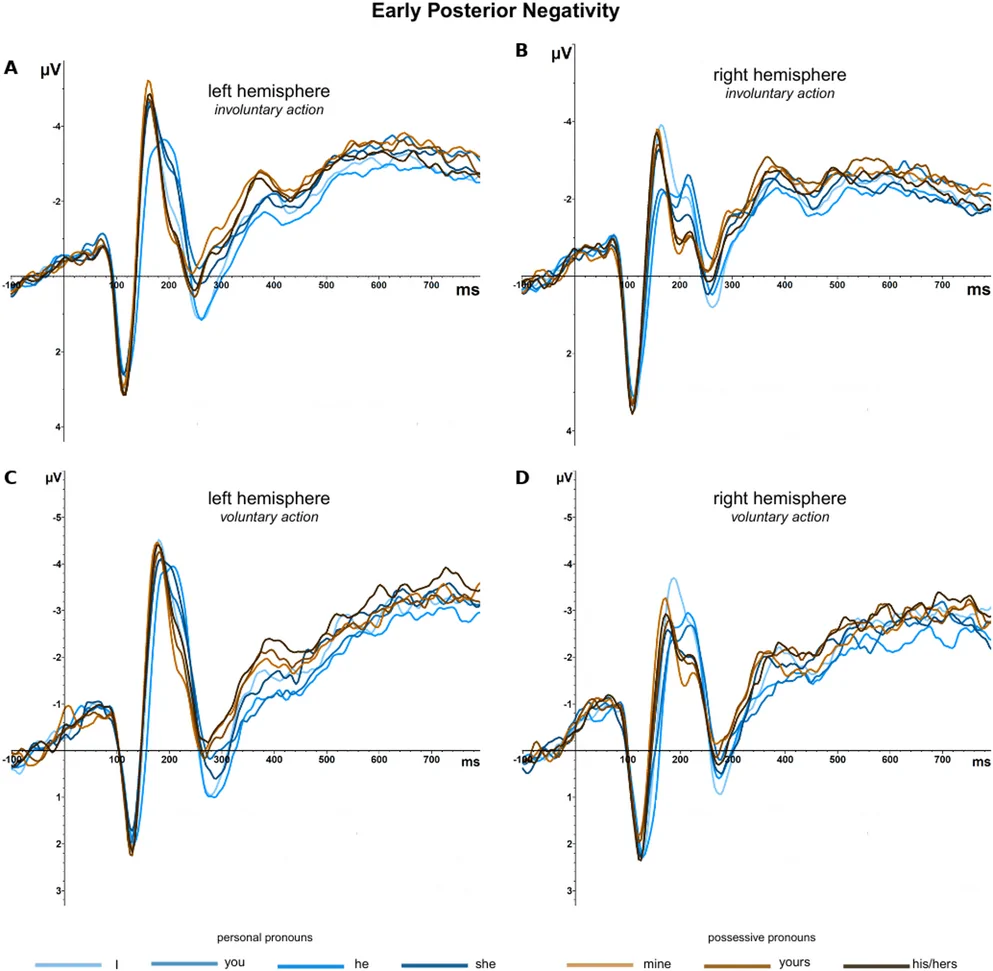
Grand-average EPN waveforms elicited by German personal and possessive pronouns at posterior-occipital electrode sites, shown separately for the involuntary and voluntary keypress conditions. Waveforms were averaged across the EPN electrode cluster and are displayed separately by hemisphere (left: PO3, PO7, O1; right: PO4, PO8, O2). (**A**) Left hemisphere, involuntary (forced) keypress. (**B**) Right hemisphere, involuntary (forced) keypress

**Fig. 5:**
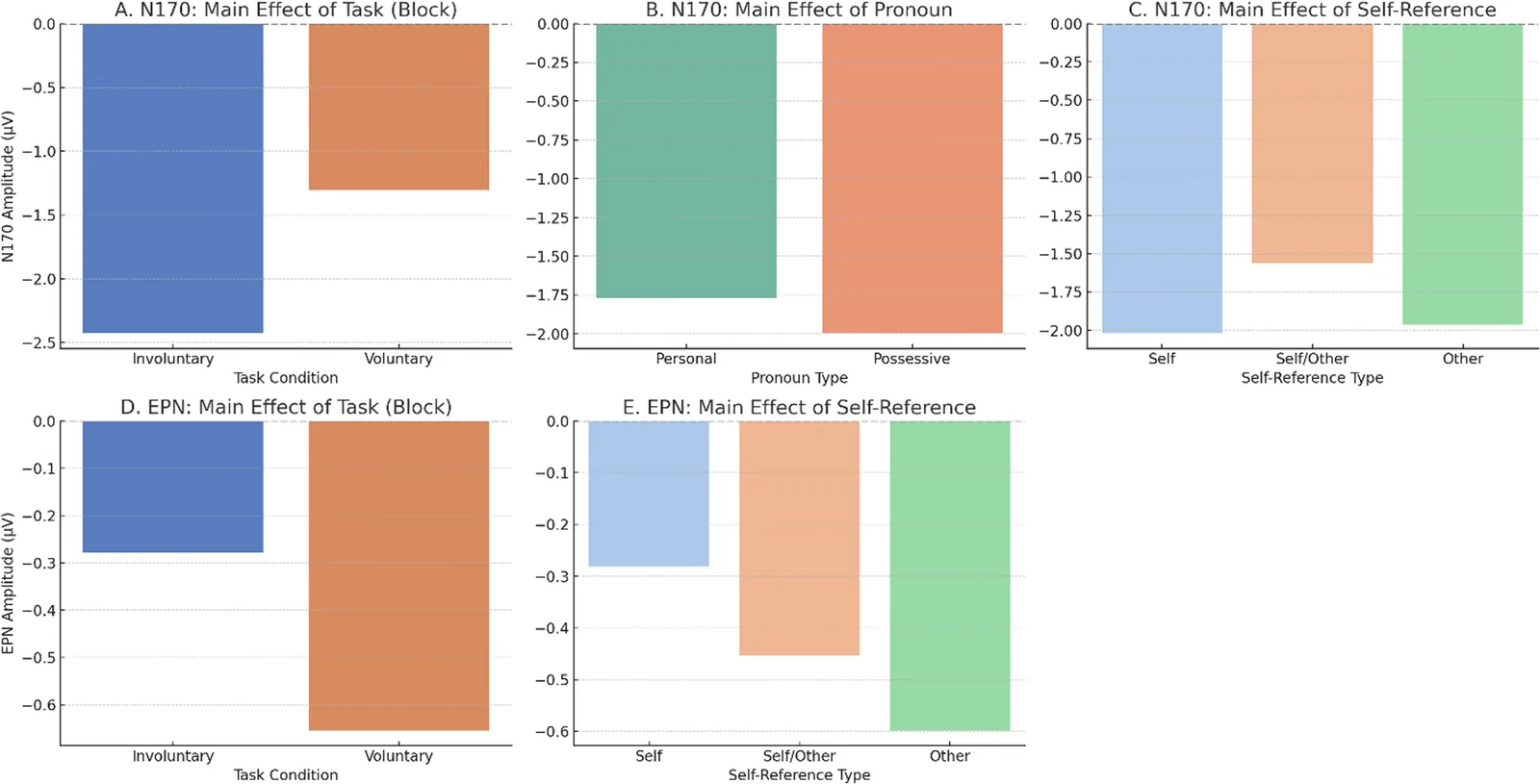
Main effects of task condition, pronoun type, and self-reference on N170 and EPN amplitudes*.* (**A**–**C**) N170 amplitudes as a function of task condition (**A**), pronoun type (**B**), and self-reference (**C**). (**D**–**E**) EPN amplitudes as a function of task condition (**D**) and self-reference (**E**)

**Fig. 6:**
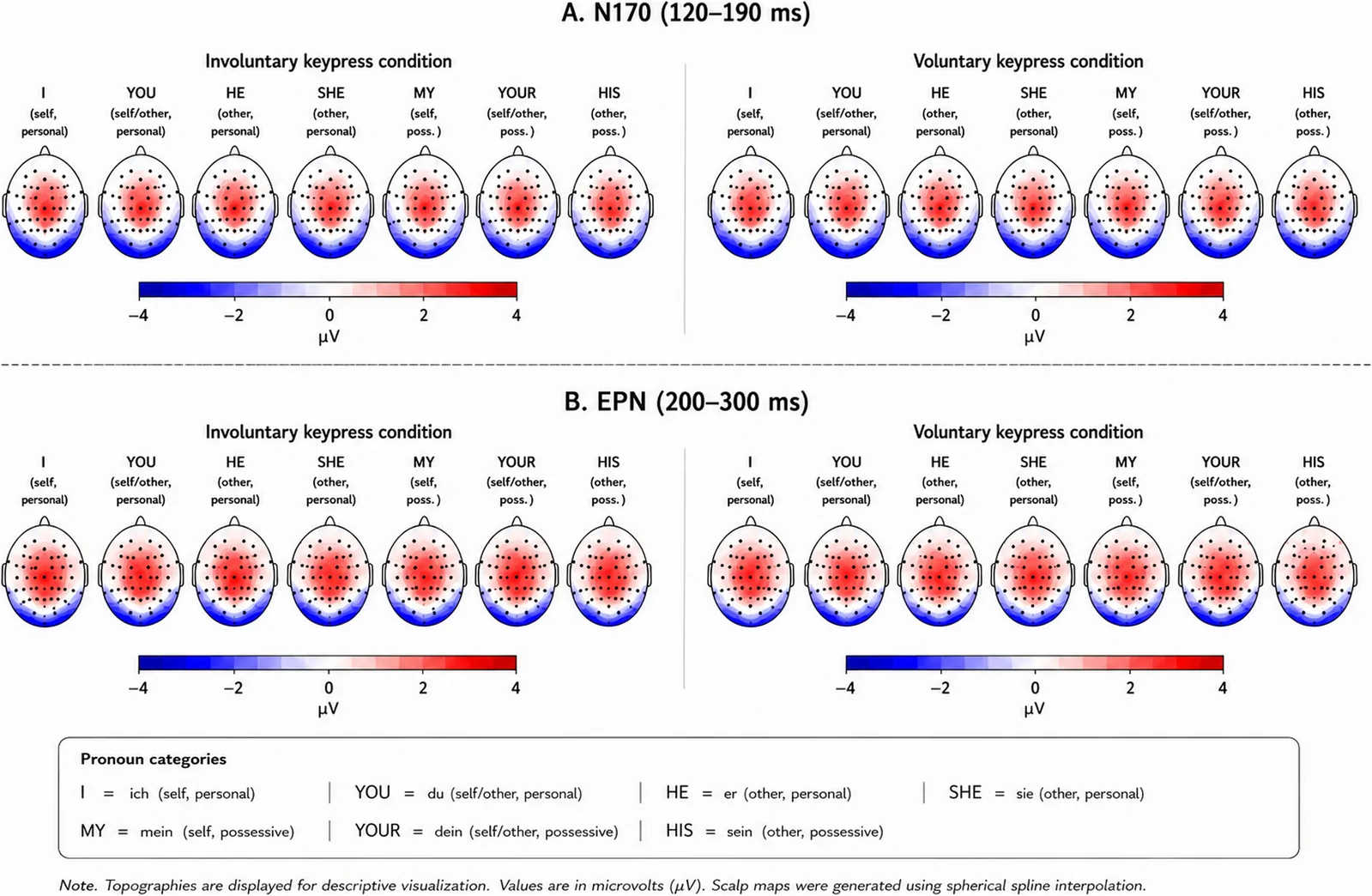
Topographic distribution of ERP activity during N170 and EPN time windows across task conditions. Topographic scalp maps illustrate mean ERP amplitudes during the N170 (120–190 ms) and EPN (200–300 ms) intervals separately for involuntary and voluntary task conditions. Maps are displayed for each pronoun category and represent averaged activity across posterior electrode regions (N170: P7, P8, PO7, PO8; EPN: PO3, PO4, PO7, PO8, O1, O2). Warmer colors indicate relatively positive amplitudes and cooler colors relatively negative amplitudes. Topographies are presented for descriptive visualization purposes

Grand-average waveforms for the N170 and EPN components are presented in Figs. 3 and 4, respectively. The significant main effects described below are additionally presented in Fig. 5. We also incorporated topographic maps that present the temporal progression of stimulus processing, beginning at stimulus onset and continuing across the entire epoch (Fig. 6).*3.1. Descriptive Behavioral Data*Descriptive behavioral response latencies indicated faster responses in the involuntary condition (M = 269 ms, SD = 134 ms) than in the voluntary condition (M = 423 ms, SD = 225 ms). This pattern is consistent with the different response requirements across task conditions, as the involuntary condition required immediate responding at stimulus onset, whereas the voluntary condition allowed more flexible response timing. The mean involuntary response latency therefore occurred within the 200–300 ms EPN interval and after the 120–190 ms N170 interval. Given the breadth of the response latency distribution (SD = 134 ms), however, a proportion of responses also fell within the N170 interval, and preparatory motor activity preceding the keypress would have begun earlier still. Temporal overlap with response-related activity was therefore more pronounced for the EPN, but was not absent for the N170. Because the present study focused primarily on stimulus-locked ERP responses, no inferential behavioral analyses were performed.

### N170–120–190 ms

A repeated-measures ANOVA conducted on N170 amplitudes revealed significant main effects of BLOCK, PRONOUN, and SELF, but not LATERALITY. BLOCK-dependent effects are reported in full below, but without functional attribution: the present design does not permit their source to be identified (see Sect. 5). The effect of BLOCK was significant, F(1,39) = 90.81, *p* <.001, indicating that the involuntary condition elicited reliably different responses than the voluntary condition, as confirmed by post-hoc comparisons (T = −9.53, *p* <.001, Hedges’ g = −0.44). This difference is statistically robust but is reported without functional attribution, because the task design involved immediate responding and the observed difference cannot be separated from overlapping motor-related and anticipatory processes. A significant main effect was also observed for PRONOUN, F(1,39) = 7.08, *p* =.011, with personal pronouns eliciting significantly different amplitudes compared to possessive pronouns (T = 2.66, *p* =.011, g = 0.12). This pattern suggests that the brain differentiates between reference to agents and reference to ownership even at early perceptual stages. Additionally, the main effect of SELF was robustly significant, F(2,78) = 33.64, *p* <.001; pairwise contrasts indicated no difference between self-related and other-related references, but both differed significantly from the combined self/other-related references (self vs. self/other: T = − 6.79, *p* <.001; other vs. self/other: T = − 8.03, *p* <.001). Thus, second-person pronouns produced distinct N170 responses, likely reflecting the additional processing demands associated with their referential ambiguity. However, no significant main effect was observed for LATERALITY, F(1,39) = 3.49, *p* =.069. Among two-way interactions, only PRONOUN × SELF was significant, F(2,78) = 8.76, *p* <.001, indicating that the influence of pronoun type varied across self-reference conditions. This suggests that personal vs. possessive distinctions are processed differently depending on whether the referent corresponds to the self, the other, or an ambiguous you/your category. All other two-way interactions involving BLOCK, LATERALITY, PRONOUN, and SELF did not reach significance (*p* >.05). Notably, a significant three-way interaction between LATERALITY, PRONOUN, and SELF emerged, F(2,78) = 5.02, *p* =.009, reflecting hemisphere-specific modulation of pronoun and self-reference interactions. This indicates that early perceptual sensitivity to pronoun meaning is not uniformly distributed across hemispheres. Finally, a significant four-way interaction (BLOCK × LATERALITY × PRONOUN × SELF) was observed, *F*(2, 78) = 5.47, *p* =.006. Because this higher-order interaction includes BLOCK, it is reported as a statistically significant pattern but is not interpreted mechanistically in the current design. Together, these effects suggest that N170 responses are sensitive to interactions among task conditions, linguistic form, self-relatedness, and hemispheric factors.

### EPN – 200–300 ms

A repeated-measures OMNIBUS ANOVA conducted on EPN amplitudes revealed significant main effects of BLOCK, F(1,39) = 6.21, *p* =.017, and SELF, F(2,78) = 6.06, *p* =.004, while main effects of PRONOUN, F(1,39) = 1.80, *p* =.188, and LATERALITY, F(1,39) = 0.60, *p* =.442, were not significant. BLOCK-dependent effects are reported in full below, but without functional attribution (see Sect. 5). Post-hoc comparisons confirmed that the involuntary task elicited significantly larger EPN amplitudes than the voluntary task (t = 2.49, *p* =.017, Hedges’ g = 0.21). This is a statistically reliable task-condition difference. Because the mean involuntary response latency occurred within the EPN measurement interval, however, it is reported without functional attribution and cannot be dissociated from response preparation, motor execution, anticipation, and other response-related activity. Pairwise contrasts within SELF indicated significant differences only between self-related and other-related references (T = 3.46, *p* =.004, g = 0.14), whereas comparisons involving self/other-related references were non-significant (ps > 0.05). This indicates that the EPN is particularly sensitive to whether the pronoun is self-referential, with clearer distinctions between self and other than between ambiguous and unambiguous categories. Among two-way interactions, significant effects were observed for BLOCK × LATERALITY, F(1,39) = 8.86, *p* =.005, LATERALITY × SELF, F(2,78) = 7.23, *p* =.001, and PRONOUN × SELF, F(2,78) = 7.15, *p* =.001. The BLOCK × LATERALITY interaction indicated that the task-condition difference varied across hemispheres. Consistent with our treatment of BLOCK-dependent effects, this interaction is reported on the basis of the omnibus test alone and is not decomposed into simple-effects contrasts. The task-condition difference varied across the left and right posterior electrode clusters; however, because the interaction includes BLOCK, no specific hemispheric mechanism is inferred. Moreover, the LATERALITY × SELF interaction was driven primarily by significant differences between self-related and other-related references (T = 4.80, *p* <.001, g = 0.20), suggesting hemisphere-specific modulation of self-processing effects. This pattern aligns with accounts of lateralized emotional and social processing. Likewise, the PRONOUN × SELF interaction showed reliable differences involving pronoun type across SELF conditions, particularly between self-related and other-related references (T = 5.35, *p* <.001, g = 0.29), and between self-related and self/other-related conditions (T = 3.26, *p* =.014, g = 0.22). Thus, semantic and referential properties of pronouns continue to influence processing during this later evaluative window. The three-way interactions BLOCK × PRONOUN × SELF, *F*(2, 78) = 6.24, *p* =.003, and LATERALITY × PRONOUN × SELF, *F*(2, 78) = 3.13, *p* =.049, were significant. The interaction involving BLOCK is reported without mechanistic interpretation because its source cannot be separated from response-timing differences. These higher-order effects suggest that EPN responses were sensitive to interactions among linguistic meaning, referential status, task condition, and hemispheric factors. No other three-way or four-way interactions reached significance (all ps > 0.05). Overall, EPN amplitudes varied with self-reference and several interactions among pronoun type, laterality, and task condition. BLOCK-dependent patterns are reported without functional attribution, because the mean involuntary response latency fell within the EPN measurement interval.

## Discussion

This study investigated how linguistic self-reference, response context, and hemispheric lateralization influence early neural responses during pronoun processing. Using an embodied cognition framework, we examined ERP markers—specifically the N170 and EPN components—in voluntary and involuntary response contexts. Our findings indicate that both components varied with pronoun type, self-relatedness, and task condition, suggesting that isolated pronouns may engage early neural processes sensitive to socially and self-relevant information. Task-condition effects were also present, but they are reported without functional attribution because the conditions differed systematically in response timing and motor demands.

### N170: Early perceptual and self-referential processing

The N170 analysis revealed significant main effects of Block, Pronoun, and Self, indicating that early perceptual processing is sensitive to task demands, lexico-semantic context, and self-referential content. To maintain consistency with how the N170 is typically interpreted, we now describe these effects solely in terms of early perceptual responses to socially meaningful or self-relevant stimuli, without invoking attentional mechanisms. The involuntary and voluntary conditions elicited reliably different N170 amplitudes. Because the conditions also differed in response timing and motor demands, this difference is reported without functional attribution and cannot be assigned specifically to agency. The posterior N170 ROI did not include central motor electrodes, which may reduce sensitivity to maximal local motor activity, but does not exclude spatially distributed response-preparatory contributions. Although the mean response latency fell after the N170 interval, the breadth of the latency distribution means that response-related activity cannot be excluded from this interval either. The differentiation between personal and possessive pronouns further indicates that linguistic form carries distinct social-cognitive weight even at early perceptual stages (Brunyé et al., 2011). Additionally, the pronounced N170 responses to the combined self/other (‘you’ and ‘your’) pronouns suggest that their referential flexibility evokes greater neural effort, likely due to conflict or integration demands inherent in distinguishing self from other (Sui & Humphreys, 2015). To clarify this further, we note that second-person forms depend on interlocutor context to determine whether they refer to self or other, which may introduce additional processing demands already at early perceptual stages. This referential ambiguity arises because second-person pronouns require the cognitive system to simulate both roles implied in communication—an addressee position (self-related) and a speaker position (other-related). Such dual-role mapping increases the integration demands placed on early perceptual systems, as the brain must reconcile competing self- and other-related interpretations. Importantly, this ERP enhancement is consistent with the possibility that isolated pronouns engage neural processes related to self–other differentiation, revealing their role in early socio-perceptual processing. Finally, the interactions between Pronoun and Self, as well as the higher-order interactions involving Laterality, highlight that the brain dynamically integrates linguistic, emotional, and contextual cues in a hemisphere-dependent manner, highlighting interactions among linguistic processing, task context, and hemispheric factors during early perceptual stages.

### EPN: Early sensitivity to socially salient language

The EPN results complemented the N170 findings by capturing subsequent stages of attention and emotional appraisal. EPN amplitudes were reliably greater in the involuntary task condition (e.g., Schupp et al., 2006). Because the mean involuntary response occurred within the EPN measurement interval, this difference is reported without functional attribution and cannot be separated from response preparation, motor execution, anticipation, and other response-related activity. Additionally, significant effects for Self revealed that self-related pronouns elicited distinct neural patterns compared to other-related ones, underscoring the salience of self-referential information during early emotional processing (Herbert et al., 2011). Although the pronouns used in the current study were emotionally neutral in semantic content, their self-referential and interpersonal nature renders them motivationally salient. Prior research suggests that stimuli relevant to the self can engage early attentional mechanisms even in the absence of overt emotional valence, particularly when they signal personal relevance or social interaction potential (Northoff et al., 2006; Sui et al., 2015). In this context, pronouns such as “I” or “you” likely activate internal models of selfhood and relational dynamics, contributing to enhanced visual-attentional processing as indexed by the EPN. The observed two- and three-way interactions further substantiate this view. The Laterality × Self and Pronoun × Self interactions suggest that emotional salience is not only influenced by lexico-semantic role but also by the referential context in which it occurs. The Block × Laterality interaction indicates that the task-condition difference varied across the left and right posterior electrode clusters. However, response-related activity may itself be lateralized, preventing a specific hemispheric interpretation of this interaction (Gainotti & Marra, 2011; Hartikainen, 2021). Taken together, these findings suggest that EPN responses are sensitive not only to linguistic form and task context, but also to socially and self-relevant aspects of pronoun processing.

### Integration with embodied cognition

Together, these findings are broadly consistent with embodied cognition accounts suggesting interactions between language, self-related processing, and socially relevant contextual factors. The modulation of early ERP components by self-related language is broadly consistent with embodied accounts of linguistic processing. Task-condition effects were also observed and are reported without functional attribution; their specific functional origin remains unresolved in the present design. The hemispheric asymmetries observed across both components further align with the idea that the left and right hemispheres contribute differentially to linguistic and emotional facets of self-processing (Shobe, 2014; Depalma & Proverbio, 2024). Importantly, these findings underscore the utility of ERPs such as the N170 and EPN in revealing the temporal unfolding of embodied language processing. In sum, this study provides preliminary neurophysiological evidence that pronoun processing is not merely linguistic but also perceptually and emotionally embodied. It highlights early ERP sensitivity to self-relevant linguistic cues and task demands, while the contribution of response context cannot be separated from response preparation, execution, and anticipation. These results contribute to a growing body of research emphasizing the embodied, interactive, and context-sensitive nature of human cognition.

## Limitations of the study and future directions

Several limitations should be considered when interpreting the present findings. First, an important methodological limitation concerns the temporal overlap between stimulus processing and motor-related activity in the involuntary task condition. Because participants were instructed to respond immediately at stimulus onset, early ERP components may partially reflect response preparation, motor execution, anticipatory processes, or temporal predictability in addition to stimulus-related perceptual processing. Consequently, effects involving the BLOCK factor cannot be interpreted as reflecting agency-specific mechanisms in an unambiguous manner. Rather, differences between voluntary and involuntary conditions likely reflect a combination of task-related cognitive, motor, and anticipatory influences. The overlap was more pronounced for the EPN, because the mean involuntary response occurred within its measurement interval, whereas the mean response occurred after the N170 interval. This difference is one of degree rather than of kind. Given the breadth of the response latency distributions (SD = 134 ms in the involuntary and SD = 225 ms in the voluntary condition), a proportion of responses in both conditions fell within the N170 interval as well, and motor preparation preceding the keypress would have begun earlier still. Response-related overlap is therefore acknowledged for both components, and BLOCK-dependent effects are reported without functional attribution for the N170 as well as for the EPN. The analyzed posterior ROIs did not include central motor electrodes, where hand-related activity is generally expected to be most pronounced. However, given the referencing scheme and the volume-conducted spread of response-related activity, posterior electrode selection cannot exclude spatially distributed motor and preparatory contributions. Accordingly, BLOCK findings are retained and fully reported as statistically reliable task-condition differences. What cannot be determined in the present design is their functional source. They are therefore reported without functional attribution rather than as evidence for agency-specific modulation. Future studies should employ delayed-response paradigms, response-locked analyses, no-response control conditions, or regression-based overlap-correction and deconvolution approaches (Smith and Kutas 2015a, 2015b; Ehinger & Dimigen, 2019) to better dissociate stimulus-related and motor-related neural activity, and should report the full distribution of response latencies rather than measures of central tendency alone. Second, the present study used a relatively small and linguistically constrained stimulus set consisting of isolated German pronouns. These stimuli necessarily differed in orthographic, lexical, and perceptual characteristics such as word length, visual complexity, and lexical frequency. Such low-level differences are known to influence early ERP components, including the N170 (Hauk & Pulvermüller, 2004). Although the observed interaction effects suggest contributions beyond purely perceptual processing, the current design does not allow complete separation of lexical-perceptual influences from self-referential or social-cognitive effects. Future studies should therefore incorporate larger and more systematically matched stimulus sets, including non-pronoun control words and item-level statistical modeling approaches. Third, the interpretation of second-person pronouns requires caution. In the present study, second-person forms were treated as self/other-related because they simultaneously invoke addressee-related self-processing and speaker-related social perspective-taking. However, pronoun reference is inherently context dependent, and isolated pronouns lack the pragmatic and conversational context necessary to fully disambiguate reference. Accordingly, the present findings should not be interpreted as demonstrating fixed neural categories of “self” versus “other,” but rather as reflecting processing differences associated with experimentally defined pronoun categories under decontextualized presentation conditions. Fourth, as an approximate sensitivity estimate for within-subject effects, the present sample size (*N* = 40) provided approximately 80% power to detect effects of about dz = 0.45 at α = 0.05. This sensitivity estimate applies to simple within-subject contrasts and does not directly characterize power for complex higher-order interactions. The four-way interaction and null findings should therefore be interpreted cautiously. Finally, the sample consisted predominantly of female participants, which may limit the generalizability of the findings across gender groups. Future studies should examine whether self-related pronoun processing differs as a function of gender, linguistic background, or sociocultural factors. Despite these limitations, the present findings provide preliminary evidence that even minimal linguistic units such as pronouns engage early neural processes sensitive to self-relatedness, linguistic form, and task context. Future work using more tightly controlled experimental designs and larger stimulus sets will be important for clarifying the respective contributions of self-reference, linguistic structure, anticipation, and motor-related processing to early ERP responses during pronoun perception.

## Data Availability

No datasets were generated or analysed during the current study.
